# OpenContami: a web-based application for detecting microbial contaminants in next-generation sequencing data

**DOI:** 10.1093/bioinformatics/btab101

**Published:** 2021-02-12

**Authors:** Sung-Joon Park, Kenta Nakai

**Affiliations:** Human Genome Center, The Institute of Medical Science, The University of Tokyo, Tokyo 108-8693, Japan; Human Genome Center, The Institute of Medical Science, The University of Tokyo, Tokyo 108-8693, Japan

## Abstract

**Summary:**

Microorganisms infect and contaminate eukaryotic cells during the course of biological experiments. Because microbes influence host cell biology and may therefore lead to erroneous conclusions, a computational platform that facilitates decontamination is indispensable. Recent studies show that next-generation sequencing (NGS) data can be used to identify the presence of exogenous microbial species. Previously, we proposed an algorithm to improve detection of microbes in NGS data. Here, we developed an online application, OpenContami, which allows researchers easy access to the algorithm via interactive web-based interfaces. We have designed the application by incorporating a database comprising analytical results from a large-scale public dataset and data uploaded by users. The database serves as a reference for assessing user data and provides a list of genera detected from negative blank controls as a ‘blacklist’, which is useful for studying human infectious diseases. OpenContami offers a comprehensive overview of exogenous species in NGS datasets; as such, it will increase our understanding of the impact of microbial contamination on biological and pathological traits.

**Availability and implementation:**

OpenContami is freely available at: https://openlooper.hgc.jp/opencontami/.

**Supplementary information:**

Supplementary data are available at *Bioinformatics* online.

## 1 Introduction

Unexpected microbes that infect or contaminate host samples can be detected by next-generation sequencing (NGS). For example, an association between microbes and human diseases was discovered by analyzing datasets derived from whole-genome and transcriptome sequencing (Kumata *et al.*, 2020; Poore *et al.*, 2020). It is noteworthy that the manipulation of eukaryotic cells risks exposure to contaminating microorganisms; thus precise detection of exogenous species in NGS data is important for avoiding erroneous conclusions (Lusk, 2014; Salter *et al.*, 2014).

To improve detection accuracy, we proposed an algorithm that investigates the genomic origin of host-unmapped reads by performing simulation and statistical tests (Park *et al.*, 2019). Here, we developed this algorithm as a web-based application, named OpenContami and built a database (DB) using analytic results from publiclyavailable NGS data. In addition, we estimated microbial genera that may originate from laboratory reagents and experimental environments; these can be used as a ‘blacklist’ by those studying, for example, infectious diseases.

OpenContami offers several advantages in terms of profiling microbial contaminants: (i) user-friendly interfaces allow users to record input data, resulting in a unique accession number, which is a precious resource for inferring correlations with experimental settings; (ii) the user can open or close records to the public domain, and share data with specific OpenContami users; (iii) the OpenContami DB manages and updates contaminants by incorporating users’ records and results from public datasets; this is used as a reference for assessing other user's data.

## 2 Implementation

**Fig. 1.**
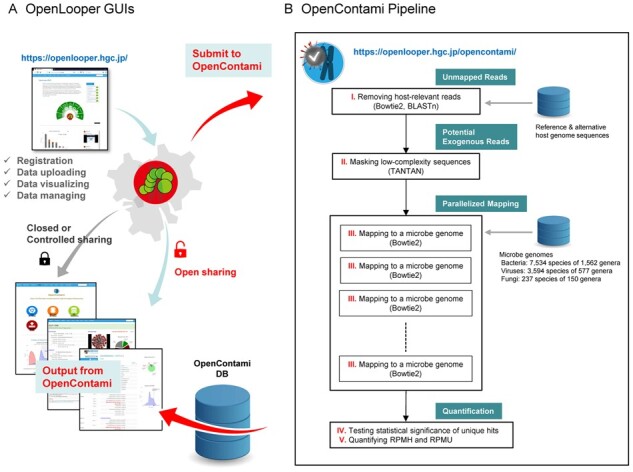
Schematic overview of the OpenContami pipeline. (**A**) Users need to upload and submit a BAM file via OpenLooper GUIs. (**B**) The pipeline conducts rigorous read mapping and statistical testing of 11 365 microbe genomes. The results are used to build the OpenContami DB. GUI, graphical user interface; DB, database; RPMH, Reads Per Million Host-mapped reads; RPMU, Reads per Million host-Unmapped reads

OpenContami has installed the pipeline, which requires huge genome sequencing and computational resources (Park *et al.*, 2019), on the supercomputer SHIROKANE using the programming language PHP and JavaScript, and the DB manager MySQL. As shown in Figure 1,one can freely register an account and handle the uploaded data via the interactive GUIs of OpenLooper (https://openlooper.hgc.jp/), an in-house repository system for NGS data.

OpenContami requests that the user uploads a BAM file that includes host-unmapped reads and inputs the total number of host-mapped reads, which is required to normalize the number of contaminant reads. Then, the application performs the pipeline by removing potential host-related reads and aligning them with 11 365 complete RefSeq genomes of bacteria, fungi and viruses. After completing the pipeline, OpenContami generates a web page describing the contamination profile, along with quantification of statistically significant genera (*P* < 0.001); data are expressed as RPMH (Reads Per Million Host-mapped reads) and RPMU (Reads per Million host-Unmapped reads). The full CSV files of the profile are downloadable from the web page (details in Supplementary Data).

## 3 Constructing the DB and blacklist

To shape the landscape of microbial contamination, we analyzed publiclyavailable human NGS read sets such as 1000 Genomes (Genomes Project*et al.*, 2015), GEUVADIS (Lappalainen *et al.*, 2013), ENCODE (Consortium *et al.*, 2020) and CCLE (Ghandi *et al.*, 2019). The results, comprising 4934 NGS read sets as of January 10, 2021, are managed as a DB that is updated continuously by incorporating more public and user-uploaded datasets. The amount of contamination present in the user data is reflected in the distribution of RPMHs in the DB to visualize how frequently this is observed.

To facilitate research into human infectious diseases, we listed genera potentially originating from reagents and the laboratory environment, all of which must be filtered out. Firstly, we analyzed 157 datasets of negative blank controls (PRJEB21503, PRJEB36408 and PRJEB7055) and merged the results with the lists identified by previous studies (Salter et al., 2014; Poore *et al.*, 2020). Then, we scored each detected genus in terms of BlackLv (black level): a higher score implies that a genus is more frequently observed in negative controls, suggesting that the target cells are not contaminated or infected by the genus (details in Supplementary Data). Of note, this list needs to be updated repeatedly as the microbial contamination can be present in both negative controls and patient samples (e.g. Salmonella in our blacklist).

## 4 Discussion

Currently, OpenContami detected 702 statistically significant microbial genera from the large-scale dataset, 192 of which are listed in the blacklist (BlackLv > 0). Since contaminants have complex characteristics, including variability across laboratories and sequencing protocols (Supplementary Fig. S4), a platform that manages contamination information based on data analysis is indispensable for establishing effective decontamination strategies. The application and the DB provide a comprehensive overview of exogenous species in data from NGS experiments, which will improve our understanding of how and why microbial species infect and contaminate host cells, as well as what impact has on the interpretation of experimental results.

## Supplementary Material

btab101_Supplementary_Data
