# Supplementary material for: OpenContami: a web-based application for detecting microbial contaminants in next-generation sequencing data
Source: Bioinformatics. 2021 Feb 12;37(18):3021–2. doi: 10.1093/bioinformatics/btab101 (PMC8479661; doi:10.1093/bioinformatics/btab101)
Supplement: btab101_Supplementary_Data [file btab101_supplementary_data.docx]

Supplementary data

**OpenContami: A Web-based Application for Detecting Microbial Contaminants in Next-generation Sequencing Data**

Sung-Joon Park* and Kenta Nakai

Human Genome Center, The Institute of Medical Science, The University of Tokyo, Tokyo 108-8693, Japan

*To whom correspondence should be addressed.

sjpark@ims.u-tokyo.ac.jp

**1. Overview of OpenContami**

OpenContami, pronounced ˈəʊp(ə)nkəntæmi: and abbreviated to OCT, maps user-uploaded NGS reads to over 11,300 microbe species genomes; we compiled [a database (DB) of microbe genomes](https://openlooper.hgc.jp/opencontami/db_microbegenome.php) based on the RefSeq complete genomes of bacteria, fungi, and viruses and developed an algorithm that exploits unique and multiple hits (Park, et al., 2019). The OCT has processed more than 4,934 publicly available NGS data sets, thereby shaping contaminant distribution. This distribution is used as a reference for user-uploaded data and is updated continuously by incorporating the analytical results of open-shared user data and public data sets. The OCT system utilizes the GUIs (graphical user interfaces) of [OpenLooper](https://openlooper.hgc.jp/) (OLP), which include email-based communication, account registration, and data manipulation. Users can request to run the OCT pipeline via OpenLooper, and the output is managed by OpenLooper (Figure S1).

The OCT quantifies microbial reads as RPMH (reads per million host-mapped reads) and RPMU (reads per million host-unmapped reads). RPMH represents the number of microbial reads mappable to known microbial genomes when a million host reads have been sequenced, while RPMU represents the number of microbial reads when a million origin-unknown reads present. These values can be calculated for a sample and for each genus. For example,

- Sample-level RPMH: “RNA-seq sample *A* has 1000 RPMH” means that when 1 million host reads sequenced in the sample *A*, 1000 reads were mapped to any microbe genomes uniquely and/or repeatedly.
- Genus-level RPMH: “Bacteria *B* in RNA-seq *A* has 1000.465 RPMH” means that when 1 million host reads sequenced in the sample *A*, 1000.465 weighted reads were found for the bacteria *B*. The weight is based on the empirical exponential scoring function (Park, et al., 2019).

Detailed information can be found from [the user’s guide](https://openlooper.hgc.jp/opencontami/help/help_oct.php).

**2. Workflow of OpenContami**

**2.1. Preparing and submitting an input BAM (Binary Alignment Map) file**

A user who has already completed [the account registration](https://openlooper.hgc.jp/register/regist1.php) process can upload a BAM file via the dashboard of OpenLooper (Figure S2). To save uploading time, it is recommended that the size of BAM files is reduced by filtering out host-mapped reads. Indeed, the OCT uses only unmapped reads by running Samtools with the SAM flag ‘-bf 4’ for the SE (single-end) input or ‘-bf 69’ for the PE (paired-end) input.

Using the annotation web page, the user can submit the BAM file to the OCT pipeline by filling in the required items (the red arrows in Figure S2). Importantly, because the BAM is recommended to include only unmapped reads, the users need to fill the total number of host-mapped reads for calculating RPMH. The total number of host-mapped reads can be counted by ‘%>samtools view -cF 4 *original_bam*’ for SE input or ‘%>samtools view -cF 132 *original_bam*’ for PE input.


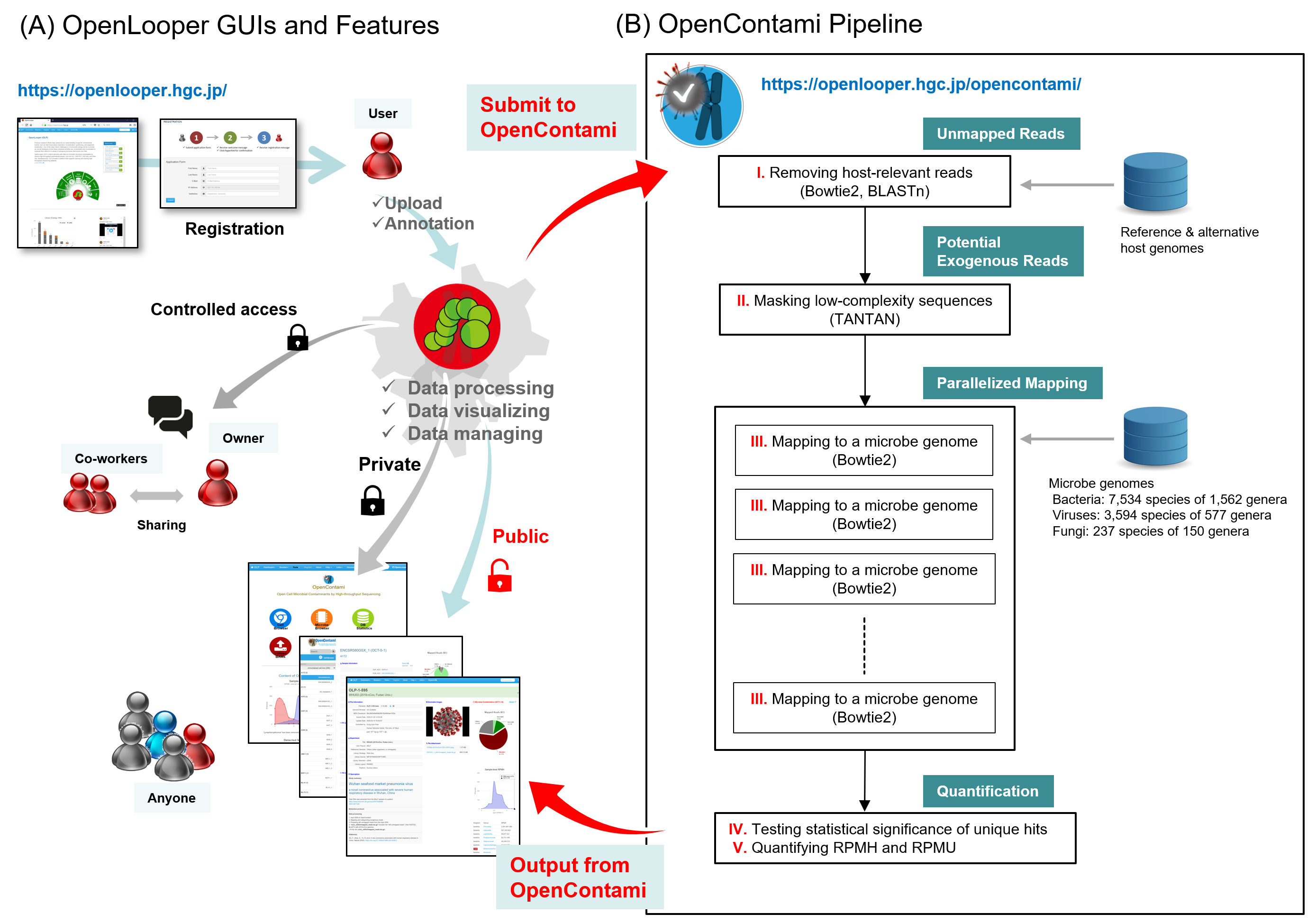


Figure S1. Schematic overview of the OpenContami (OCT) system. (A) Users can request the OCT pipeline via OpenLooper GUIs. The final outputs are managed by OpenLooper (Figure S3b), which enables easy-to-share data. (B) Step-by-step procedures in the analytical pipeline.

Note that the OCT in this current version analyzes only R1 (the first in pair) if the user input is in PE layout for reducing computational resources.

**2.2. Step-by-step procedures in the pipeline**

After running ‘%>samtools view -bf 69 *input_bam*’ for retrieving R1 reads in the case of PE reads, the following procedures are performed.

Step I (removing host-relevant reads)

To remove potential host-originated reads, the OCT performs Bowtie2 (Langmead and Salzberg, 2012) with “--sensitive” and BLASTn using the options “-evalue 0.001 -perc_identity 80 -max_target_seqs 1” sequentially. These mappers align the input reads to the reference and alternative host genomic and transcriptomic sequences available at NCBI BlastDB.

Step II (making low-complexity sequences)

The reads unmapped in Step I are candidates for exogenous reads. To reduce the false discovery rate, the OCT masks low-complexity sequences using TANTAN (Frith, 2011).


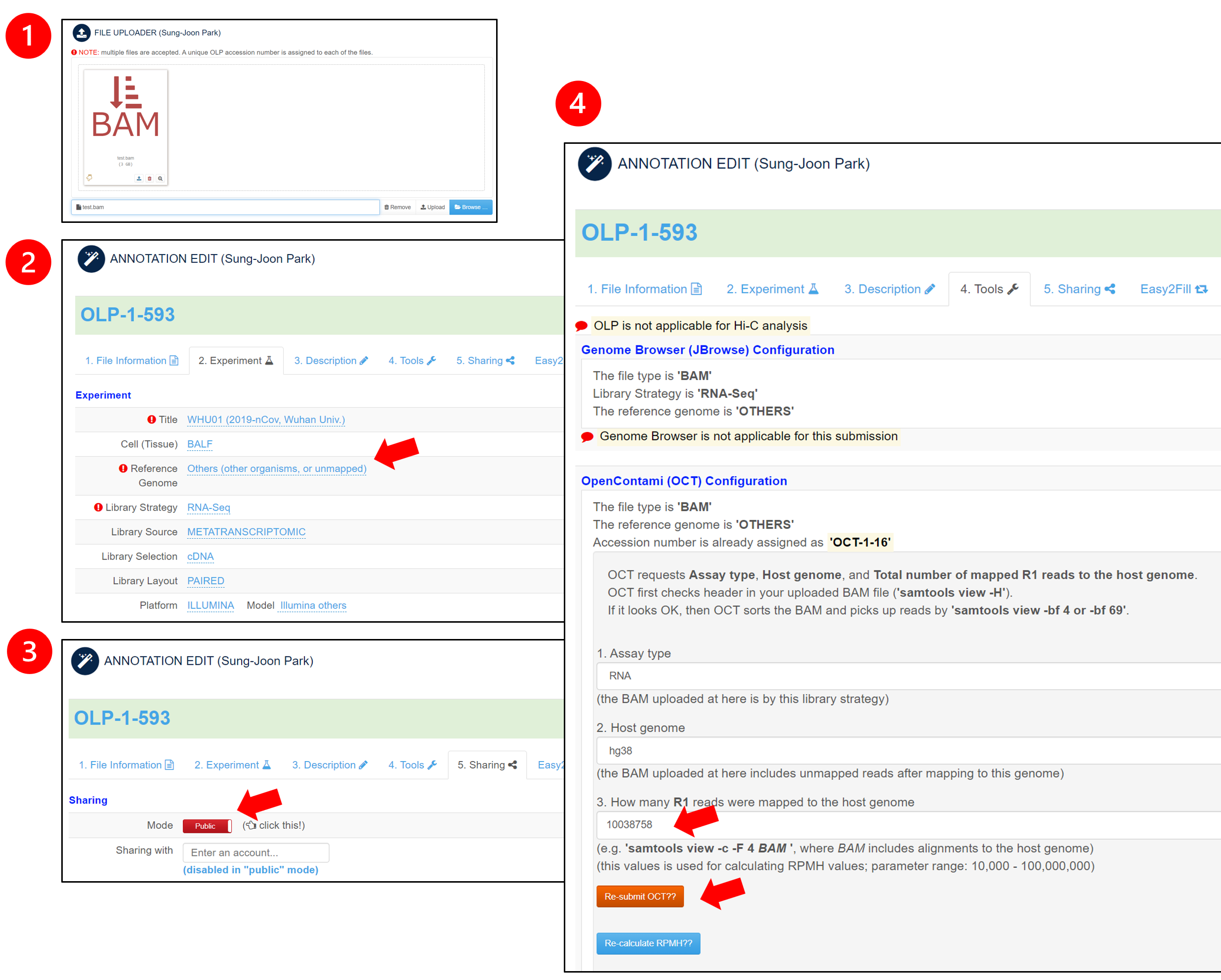


Figure S2. Four steps to submitting an input BAM file to OpenContami via OpenLooper GUIs. Users need to select “Others” as a reference genome in Step 2 and fill in the number of host-mapped reads in Step 4. The data sharing mode can be set in Step 3.

Step III (mapping to microbe genomes)

The OCT aligns the reads in Step II to one set of genomes from microbial species belonging to the same genus and parallelizes this mapping with a different genome set.

Step IV (testing the statistical significance of unique hits)

The reads mapped uniquely or repeatedly to any microbe genomes are counted as *N*. To test the chance occurrence of ‘uniq-genus-hit’ reads that were mapped to a certain microbe *G* only, the OCT randomly samples *N* reads from the microbe genome DB, excluding the *G* genome. Next, the OCT aligns these random reads to the *G* genome and counts the number of uniquely mapped reads. This procedure is repeated ten times to prepare an ensemble of random numbers of uniquely mapped reads. These numbers for *G* are converted into Z-scores, and the null hypothesis (i.e., that no difference exists between the observation of a ‘uniq-genus-hit’ and the mean of its ensemble) is tested. This test is repeated for each detected microbe genera.

Step V (quantifying RPMH and RPMU)

For sample-level quantification, the RPMH value is calculated as RPMH = *n* / *m* × 10^6^, where *n* and *m* are the total number of microbe-mapped reads and the total number of host-mapped reads, respectively. For genus-level quantification, the RPMH of a genus *G* is calculated as RPMH(*G*) = ∑S_k_ / *m*, where *k*=1,…,*t* and *t* are the total number of reads uniquely or repeatedly mapped to *G*. S_k_ is an exponentially weighted count for the read *k*.

**3. Web interface**


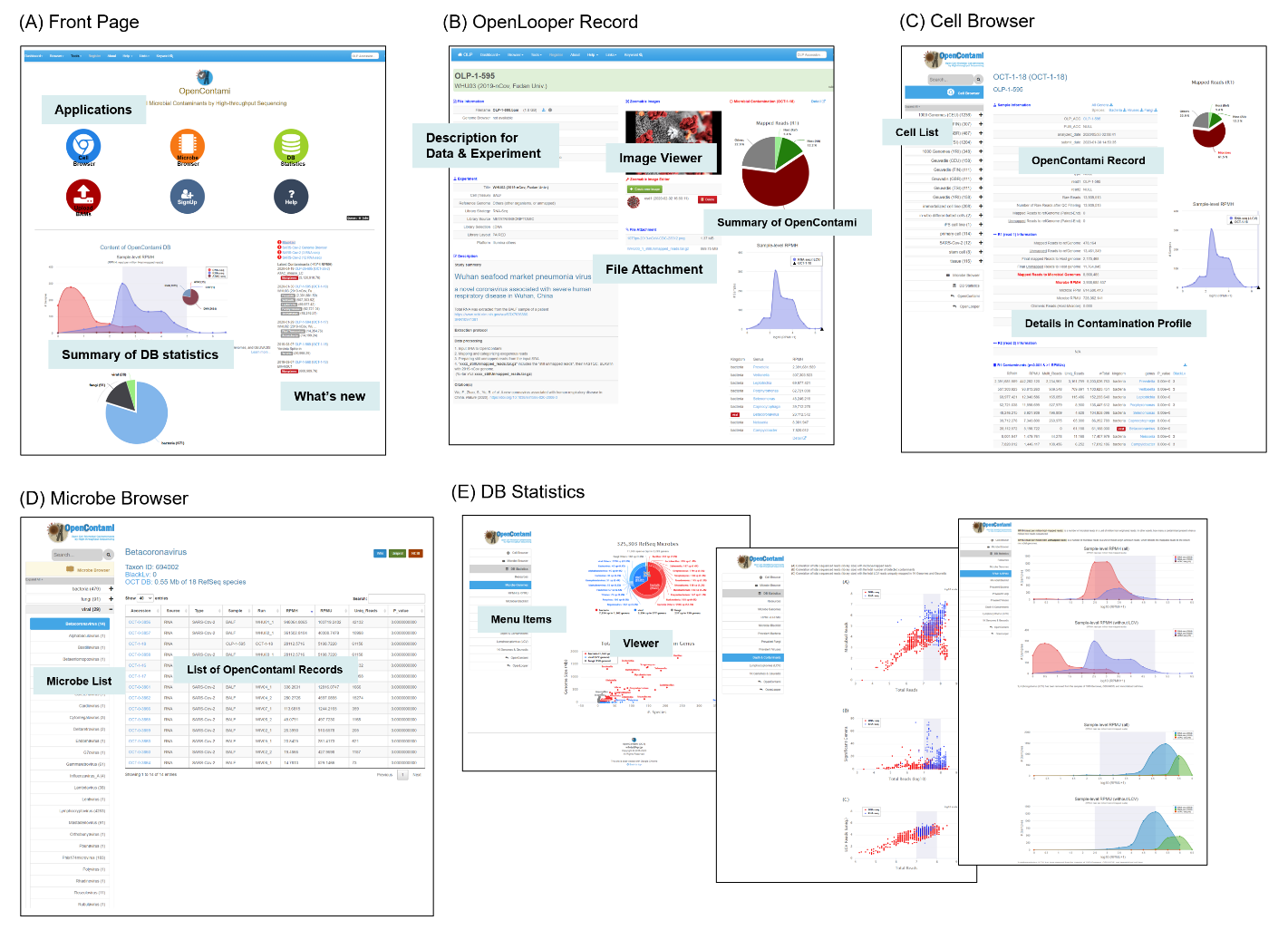


Figure S3. Snapshots of the OpenContami web interface. The unique accession numbers of OpenLooper and OpenContami are cross-linked to each other and are useful for sharing data and managing contaminant information.

Users can access the features via the OCT front page; the file upload feature is only available after login. First, OpenLooper assigns a unique accession number to the input BAM file and generates its viewer web page (Figure S3b). If the OCT succeeded in profiling the contamination, the viewer web page provides a summary of the OCT results. OCT assigns a unique accession number and generates a web page showing the details; this page is browsable via the Cell Browser feature (Figure S3c). If the data are in private mode, all the features for searching and browsing serve the owner only.

The microbes detected by the OCT pipeline are browsable via Microbe Browser and cross-referenced to the OCT records (Figure S3d). The web pages for the DB statistics summarize numbers and distributions based on DB data, which are updated frequently (Figure S3e).

**4. Processing the datasets of the 1000 Genomes Project and Geuvadis**

To characterize the microbial contamination presenting in data from NGS experiments, we analyzed the matched DNA-seq and RNA-seq samples in the 1000 Genomes Project and Geuvadis, which were sequenced at multiple laboratories. We downloaded FASTQ files for the 1000 Genomes Project (PRJNA262923) and Geuvadis (PRJEB3366) from the NCBI FTP site. The 1000 Genomes dataset is the phase 3 release of whole-genome sequences, and the Geuvadis project contains RNA sequencing (RNA-seq) data. The samples were prepared from LCLs (lymphoblastoid cell lines) immortalized by HHV4 (EBV; genus Lymphocryptovirus (LCV)). We analyzed datasets that were sequenced by both projects. Detailed information is available at <https://www.internationalgenome.org/data-portal/data-collection/geuvadis.>

Since the LCLs were infected by HHV4, we can detect LCV reads in each run and then compare reads detected in both the DNA-seq and RNA-seq assays. Overall, RNA-seq showed a higher distribution of LCV RPMHs compared to DNA-seq (Figure S4a). Also, the pairwise analysis of an LCL sample assayed by both sequencing platforms exhibited bias to RNA-seq in LCV quantity (Figure S4b). These results suggest that RNA-seq protocols present more exogenous reads, reflecting the less difference of transcriptome size than that of genome size between microbe and human.


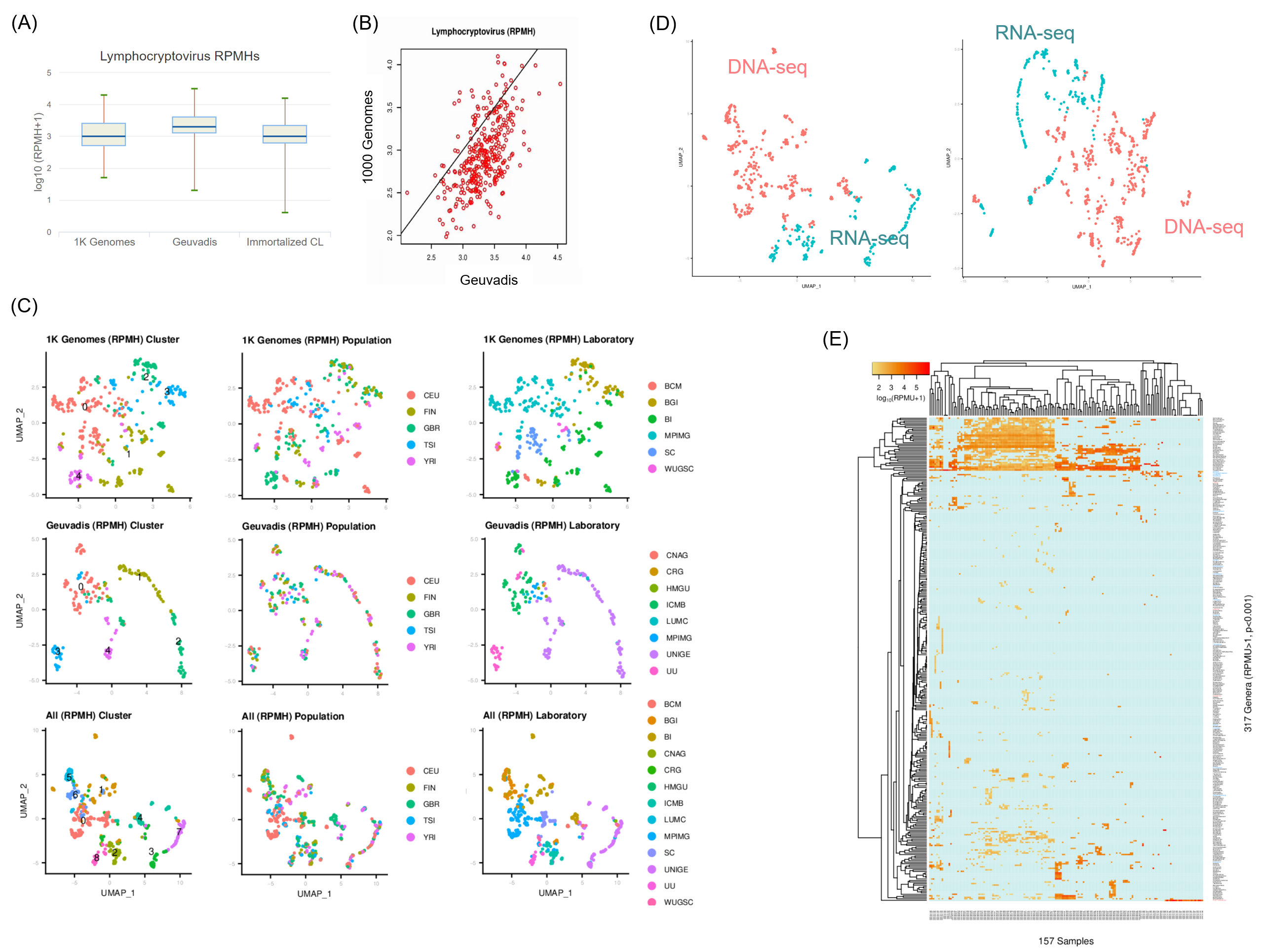


Figure S4. Analyses of sequencing samples in the 1000 Genomes, and Geuvadis, and the profile of Blank-seq. (A) Distribution of Lymphocryptovirus (LCV) RPMHs. (B) Pairwise comparison of LCV RPMHs in a sample. (C) Clustering of samples with the contamination profile of OpenContami. (D) Sample annotation of the clusters according to sequencing protocol. (E) 317 Genera identified from 157 Blank-seq samples. CL: cell line, UMAP: Uniform Manifold Approximation and Projection for Dimension Reduction.

Next, we clustered the sequencing runs using the contamination profile, excluding LCV and PhiX174microvirus (an Illumina spike-in). As shown in Figure S4c, the clusters were well correlated with the difference of sequencing laboratories (the right panel). Importantly, the sequencing protocols (i.e., DNA-seq or RNA-seq) separated the runs clearly (Figure S4d).

These results suggest that the presence of contaminants is associated with the laboratory environment, and that the contamination profiles are different between the two sequencing protocols.

**5. Generating the microbial blacklist**

Identification of contamination sources is of great importance for interpretating how detected microbes affect the biology of target cell samples. However, this is difficult because microbial contaminants present in NGS data have varying origins, including the laboratory environment, sequencer carry-over, and reagents used in DNA extraction kits. Several studies have addressed this issue using sequencing libraries prepared from ‘blank’ control samples into which no host cells were introduced; this process is named Blank-seq (Kirstahler, et al., 2018; Poore, et al., 2020; Salter, et al., 2014).

We analyzed 157 runs from publicly available Blank-seq data (PRJEB21503, PRJEB36408, PRJEB7055) (Figure S4e) and scored the detected microbes. We here integrated the blacklists shown in Table S8 in the paper by Poore et al. (PMC7500457) and Table 1 in the paper by Salter et al. (PMC4228153). The score, named BlackLv (black level), ranged from 0 to 5; if OpenContami detects a contaminant in at least two Blank-seq datasets, the pooled score is 3 points. A contaminant listed in either the Poore or Salter blacklists scores 1 point. If it is listed in both the blacklists, the score is 2 points.

Collectively, the data suggest that our blacklist places more emphasis on detection based on Blank-seq data. We used BlackLv>2 as a warning level, in which high levels of microbial contamination are likely to be derived from the environment. The full list is available at <https://openlooper.hgc.jp/opencontami/db_Blacklist.php>. This list will be updated by incorporating other negative blank data, if available.

**References**

Frith, M.C. A new repeat-masking method enables specific detection of homologous sequences. *Nucleic Acids Res* 2011;39(4):e23.

Kirstahler, P.*, et al.* Genomics-Based Identification of Microorganisms in Human Ocular Body Fluid. *Sci Rep* 2018;8(1):4126.

Langmead, B. and Salzberg, S.L. Fast gapped-read alignment with Bowtie 2. *Nat Methods* 2012;9(4):357-359.

Park, S.J.*, et al.* A systematic sequencing-based approach for microbial contaminant detection and functional inference. *BMC Biol* 2019;17(1):72.

Poore, G.D.*, et al.* Microbiome analyses of blood and tissues suggest cancer diagnostic approach. *Nature* 2020;579(7800):567-574.

Salter, S.J.*, et al.* Reagent and laboratory contamination can critically impact sequence-based microbiome analyses. *BMC Biol* 2014;12:87.
